# Dyspareunia, signs of epithelial disruption, sexual abstinence, and HIV status in female sex workers in Nairobi: a cross-sectional study

**DOI:** 10.1186/s12879-023-08572-7

**Published:** 2023-09-01

**Authors:** Bastian Fischer, Walter Jaoko, Elvis Kirui, Bernard Muture, Isaac Madegwa, Lisbeth Kageni

**Affiliations:** 1 Department of Psychiatry and Psychotherapy, Hubertusburg Hospital Wermsdorf, Husarenpark 5, 04860 Torgau, Germany; 2 KAVI-Institute of Clinical Research College of Health Sciences, University of Nairobi, 3rd Floor Wing B, P.O. Box 19676 - 00202, Nairobi, Kenya; 3 Independent scholar, P.O. Box 30197-00100, Nairobi, Kenya

**Keywords:** Sexual dysfunction, Coital bleeding, Dyspareunia, Anodyspareunia, HIV infection risk, HIV key populations, Female sex workers (FSWs), Men who have sex with men (MSM)

## Abstract

**Background:**

Epithelial trauma is a risk factor of HIV infection in men who have sex with men (MSM) and female sex workers (FSWs). Painful intercourse may be indicative of epithelial tissue disruption. Previous studies on a cohort of Kenyan FSWs established an association between prolonged sexual abstinence and late HIV seroconversion. Our research objective was to establish whether there is a relationship between HIV serostatus and signs of epithelial disruption and between HIV serostatus and sexual abstinence behaviour.

**Methods:**

Participants were selected from a Nairobi health facility. A structured questionnaire was administered to 322 FSWs, who provided data on HIV status, sexual behaviour, abstinence intervals and the level of sexual dysfunction. Sexual dysfunction scores were created using parts of the Female Sexual Function Index (FSFI-19). Additional questions addressed epithelial trauma signs. Descriptive data analysis, bivariate and multivariate logistic regression were used to describe the study population and determine factors associated with living with HIV. Potential factors influencing sexual dysfunction were assessed by FSWs via self-rating.

**Results:**

36% of FSWs reported discomfort or pain during vaginal penetration half the time. 44% noticed genital bleeding half the time. Vaginal tenderness was experienced by 70.6% half the time during or after intercourse. Variables predictive of living with HIV on multivariate analysis included a medium and high score of discomfort or pain during and following vaginal penetration (medium: AOR 2.288, p-value 0.032, 95% CI 1.075–4.871; high: AOR 3.044, p-value 0.031, 95% CI 1.110–8.348). No significant association of HIV status with past abstinence durations as reported by participants could be established in the multivariate analysis. A majority of FSWs agreed that steady partnerships (81% agreement), regularity of intercourse (74%), foreplay (72%) and lubricants (65%) alleviated dyspareunia.

**Conclusions:**

Recurrent exposure to blood during sex was highly prevalent in FSWs, as was sexual dysfunction. Complaint levels were associated with living with HIV, providing evidence that reducing sexual dysfunctions may prevent HIV transmission. Preventive initiatives may be created that address sexual dysfunction in key populations and general populations with a high HIV prevalence. Subjective assessments indicate that prevention may include the promotion of sexual intercourse regularity, foreplay, and lubricant use.

**Supplementary Information:**

The online version contains supplementary material available at 10.1186/s12879-023-08572-7.

## Background

The World Health Organization emphasizes the need to focus on HIV prevention as there are gaps in the implementation of innovations to stop HIV, viral hepatitis and sexually transmitted infections by 2030 and as “most global 2020 targets […] have been missed” [1]. Sex workers and men who have sex with men (MSM) are among the key populations considered particularly vulnerable to HIV with regard to a high risk of infection, discrimination, and stigma [2]. Hyper-endemic concentrations of new HIV infections continue to be observed in nine of Kenya’s 47 counties, while young people aged 15 to 24 years account for half of new infections in Homa Bay, Siaya and Kisumu [3]. Other sexually transmitted infections have been amplifying HIV acquisition risk especially in Nyanza province [3, 4].

While the presence of other sexually transmitted infections (STIs) is a crucial co-factor of HIV transmission [5], attempts to reduce HIV infections by treating other STIs are limited in their practicability at the population level [6–8]. Associations between intimate partner violence and HIV infection among women showed significant heterogeneity depending on the study region or country [9]. Furthermore, a systematic review of studies on intravaginal practices found no conclusive evidence for a causal influence of them on HIV spread in Sub-Saharan Africa [10]. Internationally, the scale-up of HIV testing, rapid treatment and pre-exposure prophylaxis (PrEP) led to substantial reductions in new HIV infections [11]. Long-acting antiretrovirals have recently offered a promising potential for HIV PrEP [12] and agent-based model projections have identified best scenarios achieving a 90% reduction of MSM HIV infections in Chicago by 2030 using antiretroviral treatment and long term PrEP adherence [13]. However, these best scenarios can only be realised by improving on the current prevention programmes [13], and the success of pre-exposure prophylaxis (PrEP) trials among African women was limited [14–16]. Detectable drug levels for PrEP medication fell to 31% of a sample at month 12 in a recent trial in southern Africa [17], and PrEP uptake is generally lower in African Americans, young people, and in cis- and transgender women [11]. Intimate partner violence, substance use, community violence and other structural factors can represent barriers to PrEP adoption among Black American women [18]. Similarly, PrEP use was optimal in only 41.4% of 220 female sex workers (FSWs) from Nairobi, and recent client-perpetrated emotional violence was correlated significantly with decreased PrEP use [19].

This situation justifies the scaling up of multi-pronged efforts at HIV prevention, closing implementation gaps, and securing adherence to preventive measures at the population and individual level. Current implementation gaps for the successful prevention tools may justify considering ancillary prevention approaches re-evaluating the full array of HIV risk factors in order to prevent ongoing HIV transmissions:

The starting point for the variables re-examined in this study was a cohort of initially HIV-1 seronegative FSWs in Pumwani, Nairobi, who paradoxically showed a decrease in HIV seroconversion with increasing exposure to HIV through sex work [20]. The sex workers’ age, their sexual behaviour or the presence of other STIs were not associated with persisting HIV-1-seronegativity. This led researchers to suggest the possibility of the women being resistant to HIV infection, and to link the resistance to HIV-specific cytotoxic T lymphocytes (CTL) [21]. Nearly half of the women studied had CTLs directed towards epitopes of previously defined HIV virus clades [21]. At the end of the observation, 114 FSWs of the cohort had met criteria for HIV-1 resistance by remaining HIV-seronegative and PCR-negative for at least three years while continuing sex work [20]. Eleven of them seroconverted between 1996 and 2000, which was uniquely associated with “having stopped sex work entirely for at least two months during the preceding year” and with a relative reduction of sex work by two or more clients per day in a case-control analysis involving ten HIV infection risk factors [22]. A potential explanation for the late seroconversions was seen in a diminution of HIV-1-specific CTL due to the reduction of sex work [38]. Pre-existing HIV-specific CTL had been found in six of the eleven women [22]. Evidence for CD8 + cell responses to HIV-1-CTL epitopes as a mechanism of HIV resistance had been established previously in this cohort [21, 23].

For this type of mechanism for HIV resistance, however, less frequent antigenic stimulation during the earlier observation of the cohort may not sufficiently explain the large cumulative protection of FSWs against HIV infection which was associated with starting sex work in 1985 as against 1994 [20]. At a later time point, continued antigenic exposure would have been more likely than in 1985 as the Kenyan HIV epidemic had a prevalence peak in 1995 whereas HIV prevalence was low in 1985, with a continuous increase until 1995 [24]. If the CTL hypothesis for resistance were correct and recurring antigenic stimulation required in order to maintain HIV protection, then resistance to HIV would have been more likely in later years of the HIV epidemic in Kenya. The observed cumulative 100-fold HIV protection in the cohort of FSWs associated with starting sex work early in the time span between 1985 and 1994 contradicts this expectation.

Immunological effects of the interruption of sex work were re-investigated using experimental abstinence from sex in a recent study on the Pumwani sex worker cohort [25]. While CTLs declined in FSWs living with HIV during a sex break period, FSWs showed no significant changes in systemic T-cell activation markers following the interruption of sex work, neither in the group of FSWs living with HIV nor in those living without HIV but having been involved more than seven years in active sex work nor in FSWs living without HIV and active less than three years in sex work [25]. If recurring CTL responses to HIV antigens were required for HIV protection, then some immunological difference would have to be expected in at least the second of these groups. As the explanation of resistance to HIV infection by CTL responses became less plausible for the Pumwani cohort for these two reasons, we formulated and tested an alternative hypothesis for explaining the previous results in the Nairobi sex worker cohort: Continued regularity of sexual intercourse may have been a principle of avoiding HIV infection due to the recurring vaginal epithelial dilation, which may have reduced the likelihood or extent of epithelial disruption. After periods of sexual abstinence, however, the probability of epithelial disruption and minor injury may have increased.

In order to test the hypothesis, the relationship of HIV status with both past sexual abstinence durations and with epithelial disruption signs were studied in a group of female sex workers in Nairobi. We investigated both variables because a finding for anal dyspareunia among MSM paralleled the paradoxical, inverse relationship between abstinence from sex and HIV seroconversion in the Nairobi sex worker cohort:

While early “anodyspareunia” studies in MSM found a lack of lubrication, relaxation, or anal stimulation as well as anxiety as factors contributing to pain and discomfort [26, 27], in a risk factor analysis by Vansintejan et al. [28] using multivariate logistic regression for ten circumstantial and behavioural factors, only age and the frequency of sex with a partner turned out to be significantly associated with anodyspareunia. Higher frequency of anal sex was associated with less pain.

Sexual dysfunctions, especially dyspareunia, are understudied in HIV key populations. Psychological contributing factors play a role for anal dyspareunia [29]. Cognitions related to a loss of control of one’s own body and the situation during receptive sexual intercourse have been associated with worse painful sensations and distress while participants subjectively located the pain at the anal opening (51%) and inside the anus (50%) [29]. Painful intercourse for the recipient partner may be indicative of epithelial tissue being pressured, possibly causing traumatic breaches in the tissue. While there are several anatomical and microbiological differences between rectum and vagina, the anal canal below the pectinate line and the vagina are both lined with stratified squamous epithelium [30, 31] and disruptions in the epithelial barrier facilitate viral penetration and increase the efficiency of HIV infection both vaginally and anorectally [32, 33]. While some viral interactions with oral, intestinal, and genital epithelia may actively lead to microscopic disruption of epithelial junctions [34], in a large-scale clinical trial in Durban, direct evidence of vaginal epithelial disruption was recently associated with the highest risk for HIV acquisitions in women compared with abnormal vaginal discharge, genital irritation signs, and genital ulcers [33].

Additionally, indicators of epithelial trauma have been studied: In South Africa, three studies found a high prevalence of genital coital bleeding [35–37] and a prospective study found a significant association between coital genital bleeding and HIV seroconversion [38]. A multivariate analysis of behavioural, psychological, and medical risk factors showed that anal bleeding during sex affected a third of Mexican MSM at least sometimes, and that it was significantly associated with living with HIV [39]. Associations between anorectal trauma, or indications of it, and HIV seropositivity had been previously found in one other cross-sectional study [40] and in two prospective studies [41, 42]. In a more recent study, 42% of MSM subjects in Senegal reported experiencing bleeding and discharge from the anus, and 22% having anal sores or tags [43]. In South Africa, 60% of MSM subjects surveyed reported experiencing some form of rectal trauma [44]. Using conditional logistic regression models, rectal trauma with bleeding in the last 6 months was found to be a risk factor for HIV infection among MSM in Yunnan [45]. An eminent role of the biological transmission risk of anal intercourse for the spread of HIV among MSM has been established using agent-based network simulation models [46].

## Current study

The study examined the association of the frequency of sexual intercourse and abstinence gaps with HIV status and that of dyspareunia and epithelial disruption signs with HIV status in FSWs in Kenya in order to investigate these factors for potential prevention programmes against HIV and STI transmission. The size of this hidden population for HIV prevention has been estimated as 29,494 FSWs living in Nairobi [47]. The biological role of dyspareunia as potentially increasing HIV infection risk was its possible indication of epithelial anogenital trauma, an accepted HIV transmission risk [32, 33]. Various additional behavioural and individual HIV infection risks were considered in order to rule out confounding variables. Sex workers were asked to assess factors contributing to and protecting against dyspareunia.

## Methods

### Study design

A cross-sectional study design was used in a facility in Nairobi that offers HIV prevention services for female sex workers (FSWs). The study population included FSWs in this health facility.

### Study site

The study was carried out at Bar Hostess health facility, in Makadara Sub-County. Bar Hostess health facility is a non-governmental organization medical clinic, serves FSWs and has about 10,000 FSWs enrolled. It is regulated by the Kenya Medical Practitioners and Dentists Council. The facility provides comprehensive health services and offers biomedical and structural prevention. Services at the facility include HIV testing and counselling, condom distribution, HIV care and treatment.

### Study population

Inclusion criteria were identifying as a FSW, being aged 18 years and above and attending HIV services at Bar Hostess health facility.

### Sampling procedures and sample size

The Bar Hostess facility was purposively selected due to its past linkage with the University of Nairobi programme on HIV key populations. Enrolment registers were utilised in identification of active patients within the last quarter prior to data collection. Random sampling of actively enrolled clients was applied to obtain the desired calculated minimum sample size. Using the Cochran formula, this was estimated as 320 FSWs based on an HIV prevalence of 29.6% [48], at a 95% confidence level and level of precision 0.05.

### Data collection

A close-ended structured questionnaire was administered to actively enrolled clients. The questionnaire was pretested before actual data collection and necessary corrections were made. Questionnaires were partly self-administered and partly interviewer-administered. The interviews were done in English, the written version of them was passed by the Ethics and Research Committee. Five pairs of participants with self-administered questionnaires had given identical answers for all items, including age, number of births, and abstinence intervals. We assumed that they misunderstood the individual nature of the questionnaires and, possibly due to the strong sense of social cohesion, spontaneously cooperated with completion of the questionnaire without being noticed by study staff. These ten FSWs were excluded from the analysis. One FSW who reported to be 17 years of age and one FSW who had reported secondary education level and two births but refused to specify her age were excluded from the study.

### Measures

Demographic information included gender, age, and education level. HIV serostatus was self-reported while the participants had previously been tested, examined, and/or treated at the health facility.

#### Measurement of dyspareunia and signs of epithelial disruption scores

The questionnaire included six questions of the Female Sexual Function Index (FSFI-19) [49]. The first three addressed satisfaction with (i) emotional closeness, (ii) with the sexual relationship(s), and (iii) with overall sexual life in five grades (“very satisfied” to “very dissatisfied”). The specific questions on arousal or desire, lubrication, and climax from the FSFI-19 were dropped for the sake of simplicity and since they were not directly related to signs of trauma. The last three questions of the FSFI-19 directly addressed the frequency (“always or almost always” to “almost never or never”) of discomfort and pain both (iv) during and (v) after vaginal penetration as well as (vi) the degree of discomfort or pain (“very high” to “very low or none at all”). We added two questions directly aiming at physical symptoms of epithelial disruption asking about (vii) the frequency (“always” to “never”) of any notice of blood on the genitals of the sexual partners not related to menstruation and about (viii) the frequency level of vaginal itching or sensitivity during or after receptive intercourse.

#### Intercourse frequency and gaps of abstinence

Frequencies of intercourse and intervals of abstinence questions were included in the questionnaires. The number of receptive sexual intercourse events in the previous month was inquired for. Use of sex-intended objects or sex toys the size of a penis were also counted as a receptive contact. Participants reported the longest time gaps (in days) between any instances of receptive vaginal intercourse (or comparable object insertion) in the previous month. The previous month was selected because memory of the past few weeks of sexual behaviour seemed more reliable than a general subjective estimate of usual maximum abstinence gaps. The measure was a surrogate to approximate average abstinence durations. To approximate previous abstinence habits and phases, participants were asked to specify the longest abstinence gaps in their sexually active life from memory; both including times of illness, a lack of potential partners, or other reasons for involuntary abstinence, and excluding involuntary reasons for abstinence such as partner availability, illness or disability affecting sexual life at the time.

#### Sexual behaviour and individual variables for FSWs

Based on the Belgian anodyspareunia study [28], questionnaires for FSWs included the age of sexual debut, relationship status, use of water- or silicone-based lubricants, stimulation before intercourse, the number of different sex partners in a month, use of condoms, participation in group sex and having anonymous sex. Specific factors for women practising sex with men such as the number of vaginal births and engaging in intravaginal practices (insertion of herbs, potions, powders or cleansing agents for dry and tight intercourse or vaginal cleansing) were included in the questionnaire. The questionnaire inquired for the approximate duration of sexual intercourse, the use of sex toys, any history of other STIs, and participation in sado-masochistic practices as further potential risk factors of HIV infection or epithelial disruption. Information on the use of several types of drugs including stimulants, inhaled nitrites, painkillers, and alcohol in relation to sex was sought. These variables were included to examine confounding factors with epithelial injury signs and subjective complaints.

#### Subjective assessment on dyspareunia factors

In addition to behavioural and individual variables, we asked FSWs to assess the impact of the following circumstances, habits and behaviour for increasing or decreasing discomfort and pain during sex: having a steady partner, higher number of sex partners, several partners at a time, higher age, longer duration of sex, increasing regularity or frequency of intercourse, stimulation or foreplay, lubrication or personal lubricant use, condom use, sex toy insertion before sex, and sex drugs or alcohol use. For every item, FSWs judged whether dyspareunia occurred less or more, whether the item made no difference, or they abstained from judgement.

### Statistical analyses

The data were analysed using STATA 13 statistical software. Descriptive analysis was employed to obtain frequencies and measures of central tendency primary means. Some continuous variables were grouped into categories and frequencies determined. Bivariate logistic analysis was conducted giving odds ratios and confidence intervals to determine associations of several factors with HIV status. Factors associated with HIV status on bivariate analysis with a p-value of < 0.1 were included in a multivariate logistic regression to identify predictive factors for HIV status. Internal consistency was calculated for the sexual dysfunction items (i)-(viii), (i)-(iii) and (iv)-(vi) using Cronbach’s alpha. A Spearman-Brown prophecy coefficient was used for sexual dysfunction items (vii)-(viii). Subjective assessments of factors for sexual dysfunction were given as percentages of the four assessment options selected out of the total sample ratings.

### Ethical consideration

Ethical approval was granted by the Kenyatta National Hospital‒University of Nairobi Ethics and Research Committee (KNH-UoN ERC) prior to initiation of the study. The study participants were assured of confidentiality of the information they gave. Unique identifiers were used for anonymity. Written informed consent was obtained from each study participant prior to conducting the interviews.

## Results

### Demographics and sexual characteristics of female sex workers

A total of 320 FSWs with a mean age of 27.9 ± 6.9 (SD) years completed the questionnaire. The majority (54.4%) were in the 21–29 age category with the youngest at 18 years and oldest at 50 years. A proportion of 38.8% had attained secondary school education and 37.2% were living with HIV. In the previous month, 99.4% were sexually active and 140 (43.8%) had receptive intercourse more than 20 times within that period. Longest time gaps between receptive intercourse events were one to five days for the majority of the participants (52.5%, mean: 5.6 days). Mean age at first sex with a man was 16.1 years while the average number of vaginal births was 2 (see Table 1).

**Table 1:** Descriptive statistics: Female sex workers

	N	%	Mean (SD) Min- Max
**HIV Status**:			
Negative	201	62.8	
Positive	119	37.2	
**Education level**:			
Primary	74	23.1	
Secondary	124	38.8	
Tertiary	86	26.9	
University	36	11.2	
**Age category (Years)**:			27.9 (6.9) 18–50
18–20	39	12.2	
21–29	174	54.4	
30–39	78	24.4	
40–50	29	9.1	
**Sexually active in previous month**:			
Yes	318	99.4	
No	2	0.6	
**No. of times had receptive intercourse last month**:			
0–10	65	20.3	19.4 (8.4) 0–31
11–20	115	35.9	
21–31	140	43.8	
**Longest time gaps between any instance of receptive intercourse (previous month) (days)**:			
0	22	6.9	5.6 (5.0) 0–30
1–5	168	52.5	
6–10	104	32.5	
11–30	26	8.1	
**Longest time gaps between any instance of receptive intercourse (sexually active life) (days)**:			
2–30	119	37.3	69.3(63.7) 2-365
31–60	69	21.6	
61–90	72	22.6	
>90	59	22.6	
**Longest time gaps between receptive intercourse for voluntary reasons (days)**:			
1–7	81	25.3	25.6 (32.2) 1-365
8–14	57	17.8	
15–30	140	43.8	
>31	42	13.1	
**Age at first sex (years)**:			
15 and below	137	42.8	16.1 (2.8) 14–28
16–18	76	23.8	
18–29	107	33.4	
**Number of vaginal births**			2 (1) 0–8
0	62	19.4	
1	100	31.4	
2	86	27.0	
3–8	71	22.3	

### Dyspareunia and signs of trauma, abstinence gaps, and other behavioural and medical variables

Table 2 shows discomfort or pain and signs of trauma scores among FSWs. 35% of female sex workers were about equally satisfied and dissatisfied with the amount of emotional closeness during sexual activity with their partner(s). Roughly a third (35.9%) were about equally satisfied and dissatisfied with their sexual relationship with their partner(s) while 41.1% were equally satisfied and dissatisfied with their sexual life. 36% of FSWs reported they experienced discomfort or pain during vaginal penetration about half of the time, with 7.5% experiencing it all the time. The level of discomfort or pain during or following vaginal penetration was rated high or very high by 23.7%. 44% of FSWs noticed blood on the vagina or the partner’s penis not related to menstruation half of the time while 7% noticed it most times or always. Itching, burning or soreness was experienced by 70.6% of FSWs about half the time during or after intercourse.

Internal consistency for the total eight item dyspareunia and epithelial disruption sign score was acceptable with Cronbach’s alpha = 0.72. Internal consistency was good for the three discomfort or pain questions (iv)-(vi) (Cronbach’s alpha = 0.82), acceptable for the three emotional and sexual relationship satisfaction items (i)-(iii) (Cronbach’s alpha = 0.74), while the two questions on direct bleeding signs and vaginal sensitivity (vii)-(viii) lacked internal consistency if separated from the total score (Spearman-Brown prophecy coefficient = 0.62).

**Table 2:** Discomfort or pain and signs of trauma among FSWs

Variable	n	%
**(i) Satisfied with amount of emotional closeness during sexual activity with partner(s)**		
Very satisfied	69	21.6
Moderately satisfied	73	22.8
About equally satisfied and dissatisfied	112	35.0
Moderately dissatisfied	30	9.4
Very dissatisfied	36	11.3
**(ii) Satisfied with sexual relationship with partner(s)**		
Very satisfied	68	21.3
Moderately satisfied	86	26.9
About equally satisfied and dissatisfied	115	35.9
Moderately dissatisfied	36	11.3
Very dissatisfied	15	4.7
**(iii) Satisfied with overall sexual life**		
Very satisfied	61	19.1
Moderately satisfied	67	21.0
About equally satisfied and dissatisfied	131	41.1
Moderately dissatisfied	38	11.9
Very dissatisfied	22	6.9
**(iv) Experienced discomfort or pain during vaginal penetration**		
Almost always or always	24	7.5
Most times (more than half the time)	52	16.3
Sometimes (about half the time)	115	35.9
A few times (less than half the time)	112	35.0
Almost never or never	17	5.3
**(v) Experienced discomfort or pain following vaginal penetration**		
Almost always or always	16	5.0
Most times (more than half the time)	34	10.6
Sometimes (about half the time)	138	43.1
A few times (less than half the time)	105	32.8
Almost never or never	27	8.4
**(vi) Level (degree) of discomfort or pain during or following vaginal penetration**		
Very High	25	7.8
High	51	15.9
Moderate	108	33.8
Low	109	34.1
Very low or none at all	27	8.4
**(vii) Noticed blood on vagina or partner’s penis during vaginal intercourse or after vaginal sex that was not related to menstruation**		
Always	2	0.6
Most times (more than half the time)	20	6.3
Sometimes (about half the time)	140	43.8
Rarely (less than half the time)	119	37.2
Never	39	12.2
**(viii) Noticed vaginal itching, burning, or soreness during or after receptive vaginal intercourse**		
Always	4	1.3
Most times (more than half the time)	36	11.3
Sometimes (about half the time)	226	70.6
Rarely (less than half the time)	41	12.6
Never	13	4.1

### Sexual behavioural characteristics among FSWs

Table 3 shows some sexual behavioural characteristics of female sex workers. The majority (56.6%) had steady relationships at the time of the study. One third used a water or silicone-based lubricant for receptive vaginal intercourse half the time and receptive vaginal intercourse usually lasted about 5–20 min for 62.5% of FSWs. Most FSWs (70.0%) never used sex toys before vaginal intercourse. The mean number of sex partners for receptive vaginal intercourse in a month was 11. The majority of the FSWs (78%) had been diagnosed with an STI at least once while 27.2% sometimes took part in group sex. About one in five (18.1%) of the FSWs used stimulant drugs in relation to sexual intercourse while 81.8% of them used alcohol sometimes or regularly when having sex.

**Table 3:** Sexual behavioural characteristics among FSWs

**Currently in a steady relationship**		
Yes	181	56.6
No	139	43.4
**Has had a steady relationship**		
Rarely or none	55	17.9
Some	167	54.2
Through most of your youth and adult life	66	21.4
Always	20	6.5
**Uses a water- or silicone-based lubricant for receptive vaginal intercourse**		
Always	17	5.3
Most times (more than half the time)	57	17.8
Sometimes (about half the time)	106	33.1
Rarely (less than half the time)	52	16.3
Never	88	27.5
**Length of receptive vaginal intercourse usually**		
Longer than 30 minutes	14	4.4
About 20–30 minutes	56	17.5
About 10–20 minutes	103	32.2
About 5–10 minutes	97	30.3
About 5 minutes	44	13.8
Seconds to a few minutes	6	1.9
**Has finger stimulation or massage of the vagina before intercourse**		
Always	45	14.1
Most times (more than half the time)	41	12.8
Sometimes (about half the time)	116	36.3
Rarely (less than half the time)	73	22.8
Never	45	14.1
**Has sex toys, plugs, or dildos inserted before vaginal intercourse**		
Always	3	0.9
Most times (more than half the time)	7	2.2
Sometimes (about half the time)	40	12.5
Rarely (less than half the time)	46	14.4
Never	224	70.0
**Uses sex toys, plugs, or dildos on self**		
Regularly	6	1.9
Sometimes	44	13.8
Rarely	36	11.3
Never	234	73.1
**Number of sex partners for receptive vaginal intercourse in a month**	Mean 10.8	
0–5	97	30.4
6–10	106	33.2
11–20	87	27.3
21–31	29	9.1
**Been diagnosed with an STI (Syphilis, Chlamydia, Gonorrhoea, Genital Herpes, Trichomoniasis, Hepatitis C, Genital or anal warts/HPV)**		
Four or more times a diagnosis of STIs	24	9.6
Three times a diagnosis of either or several of the above	29	11.7
Twice, with one or two different ones	49	19.7
Once, with two at once	42	16.9
Once, with one	105	42.2
**Uses condoms for vaginal intercourse**		
Always	80	25.0
Most times (more than half the time)	146	45.6
Sometimes (about half the time)	71	22.2
Rarely (less than half the time)	11	3.4
Never	12	3.8
**Engages in BDSM (bondage, discipline/domination, sado-masochism) practices**		
Never	291	90.9
Rarely	9	2.8
Sometimes	20	6.3
**Engages in BDSM practices that involve blood**		
Never	10	34.5
Regularly	2	6.9
Sometimes	6	20.7
Rarely	11	37.9
**Takes part in group sex**		
Regularly	15	4.7
Sometimes	87	27.2
Rarely	47	14.7
Never	171	53.4
**Has sexual intercourse with anonymous partners**		
Regularly	32	10.0
Sometimes	150	47.0
Rarely	55	17.2
Never	82	25.7
**Engages in dry sex practices or inserts herbs, potions, powders or cleaning agents in vagina**		
Regularly	53	16.6
Sometimes	168	52.5
Rarely	32	10.0
Never	67	20.4
**Uses the following drugs in relation to sexual intercourse: Stimulants (methamphetamine, mephedrone, cocaine, crack, ecstasy, etc.)**		
Regularly	8	2.5
Sometimes	50	15.6
Rarely	45	14.1
Never	217	67.8
**Uses inhaled nitrites (poppers)**		
Regularly	10	3.1
Sometimes	31	9.7
Rarely	30	9.4
Never	245	77.7
**Uses GHB (liquid G)**		
Regularly	3	0.9
Sometimes	22	6.9
Rarely	22	6.9
Never	273	85.3
**Uses sedative drugs or pain killers**		
Regularly	27	8.5
Sometimes	77	24.1
Rarely	26	8.2
Never	189	59.3
**Uses alcohol**		
Regularly	139	43.4
Sometimes	123	38.4
Rarely	31	9.7
Never	27	8.4

### Relationship between HIV status of FSWs and sexual dysfunction signs

Sexual dysfunction signs found to be significantly associated with living with HIV included: being moderately dissatisfied with the amount of emotional closeness during sexual activity with partner(s) (i) (odds ratio (OR) 4.588, p-value 0.001, confidence interval (CI) 1.842–11.432), most times experiencing discomfort or pain during vaginal penetration (OR 7.467, p-value 0.004, 95% CI 1.904–29.278), always experiencing discomfort or pain during vaginal penetration (OR 6.533, p-value 0.013, 95% CI 1.476–28.924), most times experiencing discomfort or pain following vaginal penetration (OR 9.868, p-value 0.005, 95% CI 2.010-48.462), always experiencing discomfort or pain following vaginal penetration (OR 9.722, p-value 0.011, 95% CI 1.696–55.748), high and very high degree of discomfort or pain during or following vaginal penetration (high: OR 3.519, p-value 0.029, 95% CI 1.136–10.900; very high: OR 5.308, p-value 0.013, 95% CI 1.418–19.874), sometimes and most times noticing blood on the sex organs (sometimes: OR 4.196, p-value 0.001, 95% CI 1.736–10.141; most times: OR 3.740, p-value 0.031, 95% CI 1.124–12.443), and most times noticing vaginal itching, burning or soreness during or after vaginal sex (OR 13.412, p-value 0.018, 95% CI 1.574-114.262).

Table 4 shows results of bivariate and multivariate analyses of the relationship between HIV status of FSWs and sexual dysfunction signs, behavioural or other variables. There was no significant difference between FSWs living without HIV and FSWs living with HIV in the number of partners they had for vaginal intercourse in a month (M = 11.273, SD = 7.776 vs. M = 9.840, SD = 5.990, respectively; t(315) = 1.725, p = 0.086) nor for the number of times they had receptive vaginal intercourse in the last month (M = 19.015, SD = 8.501 vs. M = 20.193, SD = 8.101, respectively; t(316) = -1.217, p = 0.225). These two numerical variables were therefore excluded from the multivariate analysis. Items (i)-(iii) from the sexual dysfunction items for FSWs were not included as (ii) and (iii) of this cluster of general relationship items were not significantly associated with HIV infection for any of their values. All other sexual dysfunction, medical, and behavioural variables were included given that they had a p-value < 0.1 in the bivariate analysis for at least one of their values. Questions (iv)-(vi) and (vii)-(viii) were considered in their cumulative values, respectively.

Variables found to be predictive of living with HIV after multivariate analysis included a medium and high cumulative score of the frequency and level of discomfort or pain during and following vaginal penetration (items (iv)-(vi)) (medium score: adjusted odds ratio (AOR) 2.288, p-value 0.032, 95% CI 1.075–4.871; high score: AOR 3.044, p-value 0.031, 95% CI 1.110–8.348). Engaging in intravaginal practices sometimes as well as regularly significantly increased the adjusted odds of living with HIV (see Table 4). Variables found to be predictive of living without HIV after multivariate analysis included having had four to eight vaginal births, having finger stimulation and massage (foreplay) most times during sex, and having anonymous sex regularly or sometimes (see Table 4).

Later sexual debut decreased the odds of living with HIV among FSWs only in the bivariate analysis (OR 0.547, p-value 0.044, 95% CI 0.304–0.984 for first sex with 18 + years as compared to first sexual intercourse at an age of 14 or younger). Some extended ranges of abstinence gaps both in the previous month and during sexually active life also decreased the odds of living with HIV: these ranges were 15–31 days in the previous month and 61–90 days in sexually active life according to the participants’ memory (OR 0.052, p-value 0.008, 95% CI 0.006–0.464 and OR 0.511, p-value 0.042, 95% CI 0.267–0.976, respectively). These odds ratios were also not significant in the multivariate analysis. Similarly, crude odds ratios for living with HIV were significantly increased for ever having had at least one STI versus never having had an STI diagnosis although adjusted odds ratios were not whatever the history or number of previous STI diagnoses (see Table 4). Likewise, even though using sedatives and/or analgesics sometimes or regularly increased the odds of living with HIV under bivariate analysis (sometimes: OR 1.749, p-value 0.044, 95% CI 1.017–3.008, regularly: OR 2.623, p-value 0.021, 95% CI 1.157–4.944), no significant adjusted odds ratio for sedative and analgesics use was found.

**Table 4:** Bivariate and multivariate analyses of relationship between HIV status of FSWs and dyspareunia signs, sexual behaviour, medical history, drug use

Logistic regression	Crude				Adjusted			
	OR	p-value	[95% Conf	Interval]	OR	p-value	[95% Conf	Interval]
**Age first sex with man**								
0–14	1	.	.	.	1	.	.	.
15–17	0.589	0.065	0.336	1.032	0.801	0.548	0.388	1.654
18+	0.547	0.044	0.304	0.984	1.004	0.992	0.457	2.205
**Number of vaginal births**								
0	1	.	.	.	1	.	.	.
1 to 3	0.937	0.824	0.528	1.662	0.632	0.251	0.288	1.385
4 to 8	0.37	0.058	0.132	1.035	0.113	0.001	0.03	0.426
**Max. Abstinence gap in days in last month**								
0	1	.	.	.	1	.	.	.
1 to 7	0.518	0.142	0.216	1.245	0.849	0.783	0.265	2.72
8 to14	0.395	0.115	0.124	1.252	0.675	0.606	0.152	3.004
15 to 31	0.052	0.008	0.006	0.464	0.114	0.083	0.01	1.328
**Max. abstinence gap in days during sexually active life**								
0–30	1	.	.	.	1	.	.	.
31–60	0.766	0.4	0.412	1.425	0.755	0.505	0.329	1.729
61–90	0.511	0.042	0.267	0.976	0.505	0.123	0.212	1.205
91+	1.696	0.1	0.904	3.183	1.686	0.204	0.752	3.78
**Experienced discomfort or pain related to sex**								
Satisfied	1	.	.	.	1	.	.	.
Moderate	2.223	0.013	1.188	4.16	2.288	0.032	1.075	4.871
Dissatisfied	3.958	0	1.851	8.465	3.044	0.031	1.11	8.348
**Blood stain and tenderness**								
Never	1	.	.	.	1	.	.	.
Rarely/Sometimes	3.832	0.207	0.475	30.884	3.886	0.279	0.334	45.28
Most times	8.608	0.043	1.074	68.971	6.732	0.128	0.578	78.368
Always	18	0.015	1.754	184.678	11.331	0.085	0.718	178.852
**Finger stimulation and massage**								
Never	1	.	.	.	1	.	.	.
Rarely (less than half the time)	1.26	0.545	0.596	2.665	0.967	0.946	0.365	2.56
Sometimes (about half the time)	0.836	0.617	0.415	1.685	0.642	0.351	0.253	1.63
Most times (more than half the time)	0.332	0.026	0.125	0.878	0.297	0.05	0.088	1.002
Always	0.556	0.188	0.232	1.333	0.348	0.078	0.108	1.124
**Diagnosed with STIs**								
Never	1	.	.	.	1	.	.	.
Once, with one	2.589	0.007	1.299	5.162	1.992	0.122	0.831	4.774
Once, with two at once	2.297	0.053	0.988	5.344	1.079	0.893	0.356	3.276
Twice, with one or two different ones	2.364	0.037	1.053	5.311	0.972	0.958	0.336	2.812
Three times a diagnosis of either or several of the above	3.033	0.019	1.2	7.669	1.101	0.879	0.319	3.798
Four or more times a diagnosis of STIs	4.412	0.003	1.648	11.81	1.251	0.721	0.365	4.289
**Sexual intercourse with anonymous partners**								
Never	1	.	.	.	1	.	.	.
Rarely	0.964	0.916	0.487	1.907	0.664	0.349	0.282	1.564
Regularly	0.374	0.035	0.15	0.931	0.139	0.002	0.04	0.481
Sometimes	0.461	0.006	0.265	0.802	0.342	0.003	0.168	0.696
**Engages in intravaginal practices**								
Never	1	.	.	.	1	.	.	.
Rarely	1.912	0.162	0.77	4.75	1.596	0.395	0.544	4.689
Regularly	3.187	0.003	1.469	6.916	3.785	0.011	1.361	10.522
Sometimes	1.981	0.037	1.042	3.765	2.364	0.043	1.028	5.439
**Use of sedative/analgesic drugs during sex**								
Never	1	.	.	.	1	.	.	.
Rarely	0.933	0.877	0.384	2.264	0.565	0.323	0.182	1.753
Regularly	2.623	0.021	1.157	5.944	1.983	0.233	0.643	6.115
Sometimes	1.749	0.043	1.017	3.008	1.214	0.611	0.576	2.561
**Use of alcohol during sex**								
Never	1	.	.	.	1	.	.	.
Rarely	0.972	0.96	0.313	3.017	0.734	0.652	0.191	2.817
Regularly	2.147	0.093	0.881	5.232	1.127	0.831	0.374	3.403
Sometimes	0.983	0.97	0.394	2.448	0.909	0.865	0.302	2.73

### Subjective assessment of factors influencing dyspareunia

Table 5 shows the percentages of the responses given. 81% of FSWs agreed that steady partnerships lessened dyspareunia. Having had more partners eased trouble for half of the FSWs while a third thought more experience with sexual partners made no difference. Having several partners simultaneously seemed to increase sexual discomfort for 49.5%. 53% agreed that age did not play a role for pain and discomfort. 68% agreed that longer duration of vaginal intercourse led to higher discomfort. 74% agreed that higher regularity of intercourse alleviated sexual dysfunctions. Foreplay or stimulation seemed to facilitate intercourse according to 72% of FSWs. 65% agreed that lubrication or personal lubricants eased difficulties. 60% agreed that using condoms made no difference to dyspareunia. Sex toy and sex drug use were uncommon in the FSW sample although alcohol played a central role for sexual encounters. Half of the FSWs abstained from judgement for these two items. 18% said sex toys eased trouble and 20% said they made no difference. While 22% said drugs facilitated complaints, 19% said drugs had no effect on pain and discomfort.

**Table 5:** Factors influencing sexual discomfort or pain during vaginal intercourse in self-rating (FSWs)

Sexual discomfort or pain during vaginal intercourse occurs less or more...:	N	%
**With a steady relationship**		
More	9	2.8
Less	259	80.9
No difference	43	13.4
Cannot compare	8	2.5
**The more partners you already had receptive intercourse with**		
More	32	10.0
Less	156	48.5
No difference	110	34.5
Cannot compare	21	6.6
**With a higher number of partners you have sex with at a time**		
More	158	49.5
Less	55	17.2
No difference	64	20.1
Cannot compare	42	13.2
**With higher age**		
More	60	18.8
Less	44	13.8
No difference	170	53.1
Cannot compare	46	14.4
**With longer duration of intercourse**		
More	219	68.4
Less	33	10.3
No difference	46	14.4
Cannot compare	22	6.9
**With higher regularity or frequency of vaginal intercourse and/or object use**		
More	38	11.9
Less	237	74.1
No difference	35	10.9
Cannot compare	10	3.1
**After foreplay or finger stimulation before intercourse**		
More	31	9.7
Less	231	72.4
No difference	42	13.2
Cannot compare	15	4.7
**With lubrification or lubes and oils**		
More	12	3.8
Less	207	65.0
No difference	36	11.3
Cannot compare	64	20.1
**With condoms**		
More	50	15.7
Less	46	14.4
No difference	193	60.5
Cannot compare	30	9.4
**After object insertion immediately before intercourse**		
More	20	6.3
Less	57	17.9
No difference	74	23.2
Cannot compare	168	52.7
**With certain sex drugs such as GHB, alcohol**		
More	27	8.4
Less	71	22.2
No difference	62	19.4
Cannot compare	160	50.0

## Discussion

Although biomedical advancements have improved HIV prevention, there is still a need to close implementation gaps for prevention programmes and to reduce opportunities for HIV transmissions [1]. In this study, dyspareunia and signs of anogenital epithelial trauma were highly prevalent in FSWs, indicating limitations to sexual health and personal well-being. Exposure to blood during sexual encounters may increase HIV infection risk as evidenced by previous research [38, 39]. Vaginal coital bleeding in this study was more prevalent than previously described [35–37], justifying the need for aetiological analysis for possible prevention measures.

The first part of the central hypothesis linking sexual dysfunction and epithelial trauma signs to HIV infection status was confirmed in the FSW sample in so far as higher scores for dyspareunia levels and frequency were positively associated with living with HIV after multivariate analysis. There was a significant increase of the crude odds ratio of living with HIV for high scores of epithelial trauma signs, which missed significance in the multivariate analysis. The temporal direction and causal relevance of these suggested links remain debatable given the cross-sectional study design, and further investigation is needed. The relevance and utility of this cluster of subjective complaints and epithelial trauma signs for HIV risk will need to be determined by further study. Due to the nature of sex work, the items from the FSFI-19 on satisfaction with emotional closeness and with the relationship with a partner are questionable as sex workers may have merely commercial or even exploitative relationships with their partners so that the lack of an emotional connection may not be considered an aspect of sexual dysfunction in this context. In this study, the general sexual relationship questions were non-specific in their formulation and participants could refer their replies either to any steady intimate relationship or to relationships with their clients. At the time of the study, 56.6% of FSWs were in a steady relationship, and only 17.9% of FSWs had had steady relationships rarely or never. We dropped the cluster (i)-(iii) of questions on emotional closeness, relationship satisfaction and satisfaction with overall sexual life in the multivariate analysis altogether for another reason: no response category from (ii) and (iii) was significantly associated with HIV status in the bivariate analysis. Questions (iv)-(vi) concern the somatic level of complaint and should probably be preferred in future investigations. Natural lubrication and arousal questions from the FSFI-19 may be added instead.

The subjective assessment of dyspareunia factors by FSWs may imply modes of prevention for further investigation. Steady partnerships seem to be beneficial as FSWs highly agreed that discomfort occurs less with a steady partner than with casual ones. Whatever their protective mechanism, they are difficult to maintain for FSWs and other people who are promiscuous, unable or unwilling to enter steady relationships or living in social contexts favouring concurrency. As for more experience with sex partners and having several partners at once, no clear recommendation can be drawn from the sex workers’ assessment for the prevention of dyspareunia. Longer duration of intercourse may worsen discomfort so that extremely prolonged sex may be recommended against. Higher regularity of intercourse, foreplay and personal lubricant use may be considered as protective as the women agreed that these factors may ease sexual dysfunctions. The protective maximum abstinence gaps between receptive sex appear unclear at the time of writing, and further investigation is needed. Condoms seem not to interfere as most women judged their effect on dyspareunia to be irrelevant, so their role as an effective means to HIV prevention may be upheld. Finally, sedative and analgesics use may increase HIV infection risk as seen in the inferential statistics, and there was no subjective agreement that drugs would ease dyspareunia in any way.

Contrary to the second part of our hypothesis that longer abstinence breaks subsequently lead to HIV acquisition through epithelial disruption after resuming sexual activity, abstinence breaks of 15–31 days in the previous month and of 61–90 days in FSWs’ adult life were significantly associated with living without HIV. This association may be due to some FSWs generally having a very low frequency of sexual contacts so that they may have avoided HIV acquisition through this paucity of exposures. Conversely, a considerable proportion of FSWs living with HIV might have taken fewer abstinence breaks in the previous month than at the time of their HIV acquisition, which clearly had been earlier than that. A similar vagueness regarding the time of actual HIV acquisitions as related to abstinence phases obfuscates the significant odds ratio for the abstinence of 61–90 days as estimated by FSWs from memory for all their adult life. Furthermore, these two odds ratios were not significant under multivariate analysis. Determining the role of abstinence intervals for HIV status would require determining abstinence behaviour at the time of HIV acquisition, which was impossible within this cross-sectional approach with estimated data from subjective memory.

For these reasons, the significant associations between HIV status and abstinence gaps in the previous month or in adult life in the bivariate analysis may not necessarily contradict the hypothesis of shorter abstinence gaps as protective against HIV acquisition. Nonetheless, we could not establish any confirmatory relationships. Studies confirming such a link may be difficult to set up as they would have to involve only early stage HIV infections, at a time when participants can reliably remember their sexual behaviour in previous weeks. Theoretically, *usual* break durations may play a more important role than *maximum* abstinence breaks, in which case we had been investigating the wrong type of abstinence interval. An additional correlation check after the data analysis revealed at best very weak correlations between maximum abstinence intervals and dyspareunia score items (iv)-(vi) (see supplementary file).

The potential HIV transmission risk factor of longer abstinence may be innately elusive, yet with some conceptual plausibility from the empirical background the study was based on. This elusiveness and the rather counter-intuitive notion of interruptions of sexual activity as a possible risk for HIV infection may explain why appropriate previous evidence for this risk factor is almost limited to the Nairobi female sex worker cohort findings [22]. Prolonged post-partum sexual abstinence has previously been investigated as a possible risk factor for HIV, with a positive significant association in Yaoundé for all sub-groups among women attending antenatal clinics, mixed results in Kisumu with a positive significant association only for women still married to the same husband as at the time of their last birth, and only insignificant results for women in antenatal clinics in Ndola [50]. Various mediators and confounding factors to explain these mixed results have been discussed such as partner change as the main reason for prolonged abstinence, very early ritual resumption of sex in the Luo ethnic group in Kisumu, and husbands seeking extramarital partners perinatally [50].

Apart from the central hypothesis factors, steady sexual relationships alongside sex work were common in this study, which implies the importance of regular partner testing and considering PrEP given that HIV infections within heterosexual regular partnerships and unions classically outweigh the burden of HIV infections through every other mode of exposure in Kenya [51]. Personal lubricant use was moderately popular with FSWs, indicating a possible benefit of lubricants for vaginal intercourse. This was confirmed by the sex workers’ favourable assessment of lubrication as a remedy against dyspareunia. Curiously, an additional analysis of the relationship between the use of personal lubricants and reported dyspareunia degrees revealed no significant correlation between these two variables in the sample (see supplementary file). Personal lubricants, in combination with condoms, have been recommended for MSM [52] and have found high acceptance with Black American women [53]. Intravaginal substance insertion was common and linked to an increased risk of living with HIV even under multivariate analysis, yet the large-scale impact of intravaginal practices for HIV spread in Sub-Saharan African countries seems limited as per meta-analyses [10]. While such practices have long been reported as common in various Sub-Saharan African regions [54, 55], women are commonly critical of it: The majority of Zimbabwean female respondents thought that many drying agents cause cervical or uterine cancer [56]. There is an ambiguous meaning to the term ‘dry sex’. Some speakers use it to refer to sex without discharge or excessive secretions from the vagina associated with vaginal infections [57]. It is not necessarily connoted to increased friction and roughness or to the absence of normal vaginal secretion but also to the purpose of cleansing and the prevention, or self-treatment, of vaginal infections [57]. This aspect may suggest the presence of vaginal health issues as a mediating variable for the HIV risk associated with intravaginal practices. Although we did not find any multivariate links between the history of other STIs and HIV status, we did not include bacterial vaginosis among the list of possible confounding medical variables.

Painkillers and sedatives were reported to be used in moderation and alcohol use was very common among the sex workers. This behavioural factor may be a possible risk factor for HIV transmission due to reduced self-care and precaution measures under the influence of drugs and alcohol. Regular drinking increased the odds of living with HIV among FSWs but the difference was not significant. The association of analgesics and sedative drugs with HIV infection status did not reach significance on multivariate analysis. Nonetheless, HIV infection risk through risky sexual behaviour has repeatedly been linked to alcohol and drugs consumption for MSM [58, 59], for general populations with a high HIV prevalence [60, 61] and for young women [62]. Mediating variables that have been suggested include the loss of inhibitions regarding decisions around sex, a negative impact on negotiating skills for safer sex or a coincidence of intimate partner violence with heavy alcohol use. Drinking and drug use may also be a self-treatment for dyspareunia [27], and the association between living with HIV and drug use may thus be mediated via the infection risk from epithelial disruption if painful intercourse is seen as a sign of it.

We found that later sexual debut may protect against HIV infection, which is consistent with an earlier systematic review on early sexual debut as a risk factor for HIV infection [63]. Possible mediators are engagement in other risky behaviours by women who have had sex early in life or biological factors such as genital trauma [63]. In a prospective cohort study in rural KwaZulu-Natal, however, partner age disparity did not predict HIV acquisition [64]. In the current study, the link with sexual debut was not significant under multivariate analysis. The apparent protection against HIV acquisition by having anonymous partners which is implied by our cross-sectional significant associations of this behaviour with HIV status is not clear and needs further exploration. The apparent protection against HIV infection by foreplay may be explained by more relaxed tissues and better lubrication and hence less epithelial trauma, reducing the efficiency of HIV infection.

### Limitations

The study revealed significant associations among FSWs for known HIV risk-taking behaviour such as early sexual debut, intravaginal substance insertion, drug use as well as for the new links between sexual dysfunction variables and HIV serostatus. The latter, however, was self-reported, which helped to establish a trusting relationship with participants but brought less reliability and objectivity for the HIV status variable. Differences in complaint and discomfort levels may be due to a variability of disorders with lubrication or arousal, they may be related to psychological confounding factors or be linked to physiological vaginal variation, co-infections and bacterial vaginosis. Gynaecological examinations and laboratory tests were not performed, which further limits the validity of these findings, especially since the implication of epithelial disruption by painful intercourse or perceived discomfort was largely conceptual. The Nairobi population is culturally and ethnically diverse, and the role of genetics and ethnic group would need to be taken into consideration in further studies. Differences in vaginal anatomy were found between African-American and white women [65], and Frank Plummer had observed in his early HIV immunity research that many highly exposed persistently seronegative FSWs were related to each other [66].

The significant relationship between signs and symptoms of epithelial disruption and self-reported HIV infection status, as well as the FSWs’ majority assessment on sex regularity as alleviating painful intercourse, may partly be due to interviewer or confirmation bias. Interviewer-blinded ways of data collection should be aimed at in future investigations using similar sexual dysfunction scores for the validation of an associated HIV infection risk.

The aim of the current study was to search for preliminary evidence for a novel factor for HIV transmission that carried empirical and conceptual plausibility from previous HIV immunity and sexual health research. For this reason, direct implications for HIV prevention science, let alone preventive initiatives, are highly limited. The results and relationships established nonetheless justify further investigation and refinements regarding the epithelial disruption signs and sexual dysfunction scores and their association with HIV infection status or HIV acquisition. Depending on the results, this may provide further opportunities for preventive initiatives reducing HIV infection risk by lowering the level of dyspareunia and epithelial injury in key populations and general populations with a high HIV prevalence.

## Conclusions

Recurrent exposure to blood during sexual intercourse and other minor epithelial disruption signs were highly prevalent in the HIV key population sample, as was recurrent painful intercourse, which may impact overall sexual health and carry a risk for HIV transmission.

Significant associations between various aspects of sexual dysfunction and HIV infection status were found, which may be evidence that HIV transmission risk is linked to painful intercourse and to signs and symptoms of minor epithelial trauma. More foreplay, later sexual debut, and the reduction of intravaginal practices and of sedative and analgesic drug use may protect against HIV acquisition while the exact mechanisms, causal directions and cross-links of these factors require further exploration. Aetiological analysis of sexual dysfunction and epithelial trauma may help to develop innovative prevention strategies against ongoing HIV transmissions. Subjective assessments on dyspareunia factors by female sex workers indicate that prevention may include the promotion of sexual intercourse regularity, foreplay and personal lubricants after rechecking their respective protection against HIV acquisition.

We could not confirm a relationship between having longer sexual abstinence phases and HIV status within our cross-sectional method relying on subjective memory data for previous abstinence durations. Determining the role of abstinence intervals for HIV status would require determining abstinence behaviour at the time of HIV acquisition, which necessitates a longitudinal cohort study design with frequent HIV testing and sexual behaviour monitoring. Such studies may be difficult to set up.

Based on our findings, longitudinal studies on HIV infection risk may benefit from considering abstinence behaviour variables and the level of sexual dysfunction. Future studies on sexual dysfunction and HIV risk may advantageously be extended to key populations in different settings and to general populations with a high HIV prevalence, and next steps for research programmes may include examining and differentiating underlying biological factors. The study proposes novel opportunities for HIV prevention science and sexual health improvement.

### Electronic supplementary material

Below is the link to the electronic supplementary material.

Supplementary Material 1

## Data Availability

The datasets used and analysed during the current study are available from the corresponding author on reasonable request.

## List of Abbreviations

MSM: Men who have sex with men
STI: Sexually transmitted infection
FSW: Female sex worker
PrEP: Pre-exposure prophylaxis
CTL: Cytotoxic T lymphocyte

## References

[1] Global progress report on HIV, viral hepatitis and sexually transmitted infections., 2021. Accountability for the global health sector strategies 2016–2021: actions for impact. Geneva: World Health Organization; 2021. Licence: CC BY-NC-SA 3.0 IGO. https://apps.who.int/iris/rest/bitstreams/1348210/retrieve. Accessed 16 February 2023.

[2] UNAIDS. Communities at the Centre -- Global AIDS Update. 2019. UNAIDS. https://www.unaids.org/sites/default/files/media_asset/2019-global-AIDS-update_en.pdf. Accessed 16 June 2021.

[3] Mojola SA, Wamoyi J (2019). Contextual drivers of HIV risk among young african women. J Int AIDS Soc.

[4] Mugo N, Dadabhai SS, Bunnell R, Williamson J, Bennett E, Baya I (2011). Prevalence of herpes simplex virus type 2 infection, human immunodeficiency virus/Herpes simplex virus type 2 coinfection, and associated risk factors in a national, population-based survey in Kenya. Sex Transm Dis.

[5] Buve A, Jespers V, Crucitti T, Fichorova RN (2014). The vaginal microbiota and susceptibility to HIV. AIDS.

[6] Hayes R, Watson-Jones D, Celum C, van de Wijgert J, Wasserheit J (2010). Treatment of sexually transmitted infections for HIV prevention: end of the road or new beginning?. AIDS.

[7] Ward H, Rönn M (2010). Contribution of sexually transmitted infections to the sexual transmission of HIV. Curr Opin HIV AIDS.

[8] Cohen MS, Council OD, Chen JS (2019). Sexually transmitted infections and HIV in the era of antiretroviral treatment and prevention: the biologic basis for epidemiologic synergy. J Int AIDS Soc.

[9] Li Y, Marshall CM, Rees HC, Nunez A, Ezeanolue EE, Ehiri JE (2014). Intimate partner violence and HIV infection among women: a systematic review and meta-analysis. J Int AIDS Soc.

[10] Hilber AM, Francis SC, Chersich M, Pippa S, Redmond S, Bender N (2010). Intravaginal practices, vaginal infections and HIV acquisition: systematic review and meta-analysis. PLoS ONE.

[11] Buchbinder SP, Liu AY (2019). CROI 2019: advances in HIV prevention and plans to end the epidemic. Top Antivir Med.

[12] Agrahari V, Anderson SM, Peet MM, Wong AP, Singh ON, Doncel GF, Clark MR (2022). Long-acting HIV pre-exposure prophylaxis (PrEP) approaches: recent advances, emerging technologies, and development challenges. Expert Opin Drug Deliv.

[13] Vermeer W, Gurkan C, Hjorth A, Benbow N, Mustanski BM, Kern D (2022). Agent-based model projections for reducing HIV infection among MSM: Prevention and care pathways to end the HIV epidemic in Chicago, Illinois. PLoS ONE.

[14] Van Damme L, Corneli A, Ahmed K, Agot K, Lombaard J, Kapiga S (2012). Preexposure prophylaxis for HIV infection among african women. N Engl J Med.

[15] Marrazzo JM, Ramjee G, Richardson BA, Gomez K, Mgodi N, Nair G (2015). Tenofovir-based preexposure prophylaxis for HIV infection among african women. N Engl J Med.

[16] Celum CL, Delany-Moretlwe S, McConnell M, Van Rooyen H, Bekker LG, Kurth A (2015). Rethinking HIV prevention to prepare for oral PrEP implementation for young african women. J Int AIDS Soc.

[17] Celum C, Hosek S, Tsholwana M, Kassim S, Mukaka S, Dye BJ (2021). PrEP uptake, persistence, adherence, and effect of retrospective drug level feedback on PrEP adherence among young women in southern Africa: results from HPTN 082, a randomized controlled trial. PLoS Med.

[18] Nydegger LA, Dickson-Gomez J, Ko TK (2021). Structural and syndemic barriers to PrEP adoption among black women at high risk for HIV: a qualitative exploration. Cult Health Sex.

[19] Leis M, McDermott M, Koziarz A, Szadkowski L, Kariri A, Beattie TS (2021). Intimate partner and client-perpetrated violence are associated with reduced HIV pre-exposure prophylaxis (PrEP) uptake, depression and generalized anxiety in a cross-sectional study of female sex workers from Nairobi, Kenya. J Int AIDS Soc.

[20] Fowke KR, Nagelkerke NJ, Kimani J, Simonsen JN, Anzala AO, Bwayo JJ (1996). Resistance to HIV-1 infection among persistently seronegative prostitutes in Nairobi, Kenya. Lancet.

[21] Rowland-Jones SL, Dong T, Fowke KR, Kimani J, Krausa P, Newell H (1998). Cytotoxic T cell responses to multiple conserved HIV epitopes in HIV-resistant prostitutes in Nairobi. J Clin Invest.

[22] Kaul R, Rowland-Jones SL, Kimani J, Dong T, Yang HB, Kiama P (2001). Late seroconversion in HIV-resistant Nairobi prostitutes despite pre-existing HIV-specific CD8 + responses. J Clin Invest.

[23] Kaul R, Plummer FA, Kimani J, Dong T, Kiama P, Rostron T et al. HIV-1-specific mucosal CD8 + lymphocyte responses in the cervix of HIV-1-resistant prostitutes in Nairobi. J Immunol. 2000 Feb. 10.4049/jimmunol.164.3.1602 10640781

[24] Awad SF, Abu-Raddad LJ (2014). Could there have been substantial declines in sexual risk behavior across sub-saharan Africa in the mid-1990s?. Epidemics.

[25] Omollo K, Boily-Larouche G, Lajoie J, Kimani M, Cheruiyot J, Kimani J et al. The impact of sex work interruption on blood-derived T cells in sex workers from Nairobi, Kenya. AIDS Res Hum Retroviruses 2016 Oct/Nov;32(10–11):1072–8. 10.1089/aid.2015.0332 PMC5067831 26879184

[26] Rosser BR, Short BJ, Thurmes PJ, Coleman E (1998). Anodyspareunia, the unacknowledged sexual dysfunction: a validation study of painful receptive anal intercourse and its psychosexual concomitants in homosexual men. J Sex Marital Ther Oct-Dec.

[27] Damon W, Rosser BR (2005). Anodyspareunia in men who have sex with men: prevalence, predictors, consequences and the development of DSM diagnostic criteria. J Sex Marital Ther Mar-Apr.

[28] Vansintejan J, Vandevoorde J, Devroey D (2013). The GAy MEn sex StudieS: Anodyspareunia among belgian gay men. Sex Med.

[29] Yessick LR, Gauvin SEM, Salomons TV, Pukall CF (2023). Pain characteristics, sexual script flexibility, and penetration control cognitions in those experiencing anodyspareunia. Psychol Sexuality.

[30] Mitchell AWM, Drake RL, Vogl AW. Gray’s Basic Anatomy E-Book. USA:Elsevier Health Sciences; 2016. p. 221.

[31] Devi SV (2016). Basic Histology: a Color Atlas & text.

[32] Longo DL, Fauci AS. The human retroviruses. In: Kasper DL, Fauci AS, Hauser SL, Longo DL, Jameson JL, Loscalzo J, editors. Harrison’s Principles of Internal Medicine. 19th edition. New York: McGraw Hill Professional; 2015. p. 225e.

[33] Abbai NS, Wand H, Ramjee G (2016). Biological factors that place women at risk for HIV: evidence from a large-scale clinical trial in Durban. BMC Womens Health.

[34] Tugizov S (2016). Human immunodeficiency virus-associated disruption of mucosal barriers and its role in HIV transmission and pathogenesis of HIV/AIDS disease. Tissue Barriers.

[35] Kalichman SC, Simbayi LC (2004). Sexual exposure to blood and increased risks for heterosexual HIV transmission in Cape Town, South Africa. Afr J Reprod Health.

[36] Kalichman SC, Simbayi LC (2005). Sexual exposure to blood and behavioural risks among STI clinic patients in Cape Town, South Africa. Sex Health.

[37] Kalichman SC, Simbayi LC, Cain D, Cherry C, Jooste S (2006). Coital bleeding and HIV risks among men and women in Cape Town, South Africa. Sex Transm Dis.

[38] Kalichman SC, Cain D, Simbayi LC (2010). Behavioral changes associated with testing HIV-positive among sexually transmitted infection clinic patients in Cape Town, South Africa. Am J Public Health.

[39] Coplan PM, Gortmaker S, Hernandez-Avila M, Spiegelman D, Uribe-Zuniga P, Mueller NE (1996). Human immunodeficiency virus infection in Mexico City. Rectal bleeding and anal warts as risk factors among men reporting sex with men. Am J Epidemiol.

[40] Chmiel JS, Detels R, Kaslow RA, Van Raden M, Kingsley LA, Brookmeyer R (1987). Factors associated with prevalent human immunodeficiency virus (HIV) infection in the Multicenter AIDS Cohort Study. Am J Epidemiol.

[41] Darrow WW, Echenberg DF, Jaffe HW, O’Malley PM, Byers RH, Getchell JP (1987). Risk factors for human immunodeficiency virus (HIV) infections in homosexual men. Am J Public Health.

[42] Coates RA, Calzavara LM, Read SE, Fanning MM, Shepherd FA, Klein MH (1988). Risk factors for HIV infection in male sexual contacts of men with AIDS or an AIDS-related condition. Am J Epidemiol.

[43] Niang CI, Tapsoba P, Weiss E, Diagne M, Niang Y, Moreau AM (2003). It’s raining stones’: Stigma, violence and HIV vulnerability among men who have sex with men in Dakar. Senegal Cult Health Sexuality.

[44] Lane T, Shade SB, McIntyre J, Morin SF (2008). Alcohol and sexual risk behavior among men who have sex with men in South african township communities. AIDS Behav.

[45] Xu HL, Jia MH, Min XD, Zhang RZ, Yu CJ, Wang J (2013). Factors influencing HIV infection in men who have sex with men in China. Asian J Androl.

[46] Beyrer C, Baral SD, van Griensven F, Goodreau SM, Chariyalertsak S, Wirtz AL (2012). Global epidemiology of HIV infection in men who have sex with men. Lancet.

[47] Okal J, Geibel S, Muraguri N, Musyoki H, Tun W, Broz D (2013). Estimates of the size of key populations at risk for HIV infection: men who have sex with men, female sex workers and injecting drug users in Nairobi, Kenya. Sex Transm Infect.

[48] Musyoki H, Kellogg TA, Geibel S, Muraguri N, Okal J, Tun W (2015). Prevalence of HIV, sexually transmitted infections, and risk behaviours among female sex workers in Nairobi, Kenya: results of a Respondent Driven Sampling Study. AIDS Behav.

[49] Rosen R, Brown C, Heiman J, Leiblum S, Meston C, Shabsigh R (2000). The female sexual function index (FSFI): a multidimensional self-report instrument for the assessment of female sexual function. J Sex Marital Ther Apr-Jun.

[50] Glynn JR, Buvé A, Caraël M, Macauley IB, Kahindo M, Musonda RM, Zekeng L (2001). Is long postpartum sexual abstinence a risk factor for HIV?. AIDS.

[51] Gelmon L, Kenya P, Oguya F, Cheluget B, Haile G, Kenya HIV, Prevention Response. and Modes of Transmission Analysis. Final Report. Kenya National AIDS Control Council. https://icop.or.ke/wp-content/uploads/2016/09/KenyaMOT-2009.pdf. Accessed 16 June 2021.

[52] Beyrer C, Sullivan PS, Sanchez J, Dowdy D, Altman D, Trapence G (2012). A call to action for comprehensive HIV services for men who have sex with men. Lancet.

[53] Sanders SA, Crosby RA, Milhausen RR, Graham CA, Tirmizi A, Yarber WL (2018). Women’s willingness to experiment with condoms and lubricants: a study of women residing in a high HIV seroprevalence area. Int J STD AIDS.

[54] Brown JE, Ayowa OB, Brown RC (1993). Dry and tight: sexual practices and potential AIDS risk in Zaire. Soc Sci Med.

[55] Scorgie F, Kunene B, Smit JA, Manzini N, Chersich MF, Preston-Whyte EM (2009). Search of sexual pleasure and fidelity: vaginal practices in KwaZulu-Natal, South Africa. Cult Health Sex.

[56] Civic D, Wilson D (1996). Dry sex in Zimbabwe and implications for condom use. Soc Sci Med.

[57] Gafos M, Mzimela M, Sukazi S, Pool R, Montgomery C, Elford J (2010). Intravaginal insertion in KwaZulu-Natal: sexual practices and preferences in the context of microbicide gel use. Cult Health Sex.

[58] Romanelli F, Smith KM, Thornton AC, Pomeroy C, Poppers (2004). Epidemiology and clinical management of inhaled nitrite abuse. Pharmacotherapy: The Journal of Human Pharmacology and Drug Therapy.

[59] Ostrow DG, Plankey MW, Cox C (2009). Specific sex drug combinations contribute to the majority of recent HIV seroconversions among MSM in the MACS. J Acquir Immune Defic Syndr.

[60] Reid SD, Malow RM, Rosenberg R (2012). Alcohol, drugs, sexual behavior, and HIV in Trinidad and Tobago–the way forward. J Int Assoc Physicians AIDS Care (Chic).

[61] Chersich MF, Rees HV (2010). Causal links between binge drinking patterns, unsafe sex and HIV in South Africa: its time to intervene. Int J STD AIDS.

[62] Carey KB, Guthrie KM, Rich CM, Krieger NH, Norris AL, Kaplan C (2019). Alcohol use and sexual risk behavior in Young Women: a qualitative study. AIDS Behav.

[63] Stöckl H, Kalra N, Jacobi J, Watts C (2013). Is early sexual debut a risk factor for HIV infection among women in sub-saharan Africa? A systematic review. Am J Reprod Immunol.

[64] Harling G, Newell ML, Tanser F, Kawachi I, Subramanian SV, Bärnighausen T (2014). Do age-disparate relationships drive HIV incidence in young women? Evidence from a population cohort in rural KwaZulu-Natal, South Africa. J Acquir Immune Defic Syndr.

[65] Pendergrass PB, Reeves CA, Belovicz MW, Molter DJ, White JH (2000). Comparison of vaginal shapes in Afro-American, caucasian and hispanic women as seen with vinyl polysiloxane casting. Gynecol Obstet Invest.

[66] Plummer FA, Ball TB, Kimani J, Fowke KR (1999). Resistance to HIV-1 infection among highly exposed sex workers in Nairobi: what mediates protection and why does it develop?. Immunol Lett.

