# Supplementary material for: Dyspareunia, signs of epithelial disruption, sexual abstinence, and HIV status in female sex workers in Nairobi: a cross-sectional study
Source: BMC Infect Dis. 2023 Sep 1;23:569. doi: 10.1186/s12879-023-08572-7 (PMC10472738; doi:10.1186/s12879-023-08572-7)
Supplement: Supplementary file 1 — Supplementary Material 1 [file 12879_2023_8572_MOESM1_ESM.doc]

**Suppl. table 1: Rank correlation between dyspareunia score items (iv)-(vi) and abstinence gaps (FSWs)**

| *Spearman's rho*  *Number of obs*  *Sig. level* | **(iv) Experienced discomfort or pain during vaginal penetration (0-4)** | **(v) Experienced discomfort or pain following vaginal penetration (0-4)** | **(vi) Level (degree) of discomfort or pain during or following vaginal penetration (0-4)** |
| --- | --- | --- | --- |
| **Longest time gaps between any instance of receptive intercourse (previous month) (days)** | -0.101  318  0.073 | -0.087  318  0.120 | -0.111  318  0.048 |
| **Longest time gaps between any instance of receptive intercourse (sexually active life) (days)** | 0.025  317  0.662 | 0.034  317  0.542 | -0.035  317  0.538 |
| **Longest time gaps between receptive intercourse for voluntary reasons (days)** | -0.056  318  0.316 | -0.017  318  0.765 | -0.134  318  0.017 |

**Suppl. table 2: Rank correlation between dyspareunia score items (iv)-(vi) and personal lubricant use (FSWs)**

| *Spearman's rho*  *Number of obs*  *Sig. level* | **(iv) Experienced discomfort or pain during vaginal penetration (0-4)** | **(v) Experienced discomfort or pain following vaginal penetration (0-4)** | **(vi) Level (degree) of discomfort or pain during or following vaginal penetration (0-4)** |
| --- | --- | --- | --- |
| **Uses a water- or silicone-based lubricant for receptive vaginal intercourse (0-4)** | 0.068  318  0.225 | 0.055  318  0.326 | 0.025  318  0.661 |
